# Retrospective Analysis of Carpal Tunnel Syndrome: Clinical Profile, Demographics, and Risk Factors at Sohar Hospital Over a Two-Year Period

**DOI:** 10.7759/cureus.86777

**Published:** 2025-06-26

**Authors:** Vinitha Leelamani, Sreenath Shankar, Mohammed Ali Al Zoubi, Mohammed Ragab Ali, Ahmed Elawady, Ravichandran Raju

**Affiliations:** 1 Neurology, Sohar Hospital, Sohar, OMN; 2 Orthopedics, Sohar Hospital, Sohar, OMN; 3 Neurology, Mansoura University, Mansoura, EGY; 4 Neurophysiology, Sohar Hospital, Sohar, OMN

**Keywords:** carpal tunnel syndrome, clinical profile, comorbidities, electrodiagnostic findings, risk factors

## Abstract

Background

Carpal tunnel syndrome (CTS) is a common entrapment neuropathy caused by compression of the median nerve within the carpal tunnel. Despite its prevalence, the clinical profile, demographics, and associated risk factors vary across populations. In Oman, CTS is increasingly recognized as a significant health concern, particularly among individuals engaged in repetitive hand activities. This study aimed to analyze these aspects in a tertiary care setting in Oman over a two-year period, providing valuable insights into the burden of disease and its determinants.

Materials and methods

This retrospective study included 182 patients diagnosed with CTS between January 2022 and December 2023. Data on demographic variables, clinical presentations, comorbidities, occupational exposures, electrodiagnostic findings, and management outcomes were collected and analyzed. Statistical comparisons between groups were performed using chi-square tests for categorical data and logistic regression for risk factor analysis, with p-values < 0.05 considered significant.

Results

The mean age of the patients was 47.5 ± 10.3 years, with a predominance of females (172, 94.5%). Housewives constituted the largest occupational group (139, 76.4%), followed by nurses (12, 6.6%) and teachers (9, 4.9%). Bilateral CTS was present in 163 (89.6%) cases. Severe CTS was more prevalent in the dominant hand (128, 70.3%). Comorbidities included obesity (76, 41.8%), diabetes mellitus (41, 22.5%), and hypothyroidism (29, 15.9%). Electrodiagnostic studies revealed higher distal motor latencies and reduced sensory nerve conduction velocities in the right hand compared to the left. Surgical management achieved superior symptom resolution (41 (87.2%) versus 65 (48.1%)) (p = 0.007) and nighttime symptom relief (43 (91.5%) versus 72 (53.3%)) (p = 0.002) compared to conservative management. Diabetes (OR 9.26, p < 0.001), obesity (OR 3.98, p = 0.002), and repetitive occupational tasks (OR 5.61, p < 0.001) were significant predictors of severe CTS.

Conclusions

This study highlights the burden of CTS among women, particularly housewives, and underscores the impact of comorbidities and occupational exposures on disease severity. Surgical management demonstrated better outcomes than conservative approaches. Strengthening public health interventions focusing on modifiable risk factors such as obesity control and improved workplace ergonomics is recommended to reduce CTS prevalence and severity.

## Introduction

Carpal tunnel syndrome (CTS) is the most prevalent entrapment neuropathy, characterized by the compression of the median nerve within the carpal tunnel at the wrist, a confined anatomical space bordered by the transverse carpal ligament and the carpal bones. Globally, CTS affects approximately 1-5% of the general population, with studies indicating a higher prevalence in females, ranging from 7-9%, compared to males, who have an estimated prevalence of 0.6% [1,2]. Hormonal changes, particularly during pregnancy, menopause, and hormonal therapy, are believed to contribute to this gender disparity by promoting fluid retention, soft-tissue edema, and altered collagen metabolism, all of which increase intracarpal pressure [3]. CTS is most common in individuals aged 40-60 years, although cases have been reported across all age groups [4].

Occupational and lifestyle factors play a significant role in the development of CTS. Individuals engaged in repetitive wrist and hand movements, such as those working in assembly lines, textile industries, or IT sectors, are at a higher risk. Studies have shown that the prevalence of CTS among industrial workers can be as high as 15-20%, with garment industry workers and IT professionals being particularly affected due to prolonged repetitive tasks, awkward wrist positions, and inadequate ergonomic practices [5,6]. In Oman and other Gulf Cooperation Council countries, musculoskeletal disorders, including CTS, have increasingly been reported among occupational groups such as manual laborers, domestic workers, and individuals engaged in physically demanding tasks. This regional context underscores the need to explore CTS risk factors and outcomes in populations with similar occupational profiles.

Systemic conditions such as diabetes mellitus, which affects 8.9% of adults, significantly increase the risk of CTS due to neuropathic and microvascular complications [7]. Other risk factors include obesity, hypothyroidism, rheumatoid arthritis, and wrist trauma, all of which contribute to increased intracarpal pressure [8].

The clinical presentation of CTS is variable, often beginning with intermittent paresthesia, numbness, or pain in the median nerve distribution, which includes the thumb, index, middle, and lateral half of the ring fingers. Symptoms are typically worse at night, leading to sleep disturbances. Advanced stages of CTS may result in thenar muscle atrophy, weakness, and reduced grip strength, profoundly affecting the patient’s quality of life and productivity [9]. Despite these significant impacts, CTS is frequently underdiagnosed in Oman due to limited awareness, variable access to healthcare facilities, and a lack of emphasis on preventive occupational health measures [10].

Electrophysiological studies remain the gold standard for diagnosing CTS, with a sensitivity and specificity ranging from 84% to 90% [11]. Additionally, common clinical tests such as Phalen’s test, which involves wrist flexion to provoke symptoms, and Tinel’s sign, which elicits tingling upon percussion over the median nerve, are frequently used as bedside diagnostic tools. Imaging modalities such as ultrasonography and MRI have also been employed to support diagnosis, particularly in atypical cases, although their use is less common in routine clinical practice [12].

Despite the global burden of CTS, there is a paucity of data on its clinical profile, demographic distribution, and associated risk factors in Oman. Understanding these factors is crucial for devising effective preventive and therapeutic strategies. This retrospective study aimed to analyze the clinical presentations, demographic characteristics, and risk factors of CTS over a two-year period at a tertiary care center.

## Materials and methods

Study design and setting

This retrospective observational study was conducted at Sohar Hospital among patients diagnosed with CTS over a two-year period between January 2022 and December 2023. This study design was selected as it is well-suited for analyzing trends, correlations, and risk factors in an established patient cohort by utilizing existing medical records. Such a design effectively allows assessing CTS characteristics in a real-world clinical setting, particularly in a tertiary care hospital with diverse patient presentations. The study aimed to investigate the clinical profile, demographic characteristics, and risk factors associated with CTS in patients who presented to the outpatient or inpatient services of the department of neurology.

Study population

The study population consisted of all patients diagnosed with CTS during the study period. Patients were included if they met the clinical and electrodiagnostic criteria for CTS. The clinical diagnosis was based on symptoms such as pain, numbness, and paresthesia in the median nerve distribution, often exacerbated at night. The electrodiagnostic confirmation relied on nerve conduction studies showing prolonged distal motor latency (>4.2 ms) or reduced sensory nerve conduction velocity (<50 m/s) [11,12].

Additional inclusion criteria included adults aged ≥18 years to ensure symptom assessment was consistent with adult presentation patterns. Patients who were pregnant at the time of diagnosis were excluded due to the transient nature of CTS during pregnancy. Furthermore, individuals with secondary causes of neuropathy, such as brachial plexopathy, diabetic polyneuropathy, or prior surgical intervention for CTS, were excluded. The analysis did not include patients with incomplete or missing medical records. After applying these criteria, 182 patients were selected for the study.

Data collection

Data were retrieved from the hospital’s electronic medical records system and cross-verified with patient case files to ensure accuracy. Demographic details such as age, sex, occupation, and hand dominance were recorded. Clinical data included the onset, duration, and severity of symptoms, nighttime exacerbation, and associated functional limitations such as reduced grip strength or difficulty performing fine motor tasks.

Detailed medical histories were reviewed to identify risk factors, including comorbid conditions such as diabetes mellitus, obesity (BMI ≥30 kg/m²), hypothyroidism, rheumatoid arthritis, and pregnancy. Occupational history was documented, particularly repetitive wrist or hand movements associated with professions like manual labor, textile work, or computer usage. Additionally, any history of wrist trauma or prior wrist surgeries was recorded.

Data on diagnostic findings, including nerve conduction study results, were collected, focusing on parameters such as distal motor latency, sensory nerve action potential, and conduction velocities. Information on treatment modalities, including conservative management with splints, corticosteroid injections, and surgical interventions like carpal tunnel release, was also documented.

Diagnostic criteria

The diagnosis of CTS was based on a combination of clinical evaluation and nerve conduction studies. Clinical evaluation included characteristic symptoms such as pain and paresthesia in the thumb, index, middle fingers, and lateral half of the ring finger. Physical examination findings were also noted, including positive Tinel’s sign or Phalen’s test. Electrodiagnostic confirmation was based on nerve conduction study, specifically focusing on prolonged distal motor latency and reduced sensory conduction velocities in the median nerve compared to the ulnar nerve [8].

Statistical analysis

Statistical analysis was conducted using SPSS version 25.0 (IBM Corp. Released 2017. IBM SPSS Statistics for Windows, Version 25.0. Armonk, NY: IBM Corp.). Continuous variables were expressed as means with standard deviations, while categorical variables were expressed as frequencies and percentages. The Shapiro-Wilk test was employed to assess the normality of continuous variables before applying parametric tests. Comparative analysis between groups was performed using the chi-square test for categorical variables and independent t-tests for continuous variables. Associations between risk factors and CTS severity were analyzed using logistic regression, adjusted for potential confounders such as age, sex, and comorbidities, with odds ratios (OR) and 95% confidence intervals (CI) reported. Statistical significance was set at a p-value < 0.05.

Ethical considerations

The Research and Ethical Review and Approval Committee of the North Batinah Governorate approved the study (approval number: MH/DGHS/NBG:MoWCSR/25/26253). Since this was a retrospective study, patient consent was waived. Confidentiality was strictly maintained by anonymizing all patient data before analysis. Data were securely stored in password-protected systems, with access restricted to authorized personnel only, following institutional and national ethical guidelines. Data will be retained securely for five years post-study completion to allow for potential audits or future research inquiries.

## Results

This study analyzed 182 CTS patients with a mean age of 47.5 ± 10.3 years. The mean age at symptom onset was 45.2 ± 9.8 years, indicating that most patients experienced CTS symptoms in their mid-40s. Females constituted 172 (94.5%) of the cohort, with housewives (139, 76.4%) being the most common occupational group, followed by nurses (12, 6.6%), teachers (9, 4.9%), and computer operators (5, 2.7%). Right-handed dominance was observed in 175 (96.2%) patients. The mean pain severity score was 7.3 ± 1.5, and the mean symptom duration was 18.4 ± 6.7 months. Bilateral CTS was noted in 163 (89.6%) cases, with the right hand more frequently affected in 128 (70.3%). In 156 (85.7%) patients, nighttime symptoms were present, and 141 (77.5%) reported functional limitations. Key risk factors included occupational exposure 82 (45.1%), wrist trauma 27 (14.8%), and a family history of CTS 19 (10.4%). Notably, no patients with documented amyloidosis were identified in the study population. These findings underscore the significant burden of CTS, particularly among women engaged in repetitive tasks (Table 1).

**Table 1:** Demographic and clinical characteristics of patients with CTS CTS: carpel tunnel syndrome, VAS: visual analog score, SD: standard deviation

Variable	Frequency (%)/mean ± SD
Age (years)	47.5 ± 10.3
Gender	
Male	10 (5.5)
Female	172 (94.5)
Occupation	
Housewife	139 (76.4)
Nurse	12 (6.6)
Teacher	9 (4.9)
Cleaner	4 (2.2)
Computer work	5 (2.7)
Typewriter	3 (1.6)
Others	10 (5.5)
Hand dominance	
Right	175 (96.2)
Left	7 (3.8)
Pain severity (VAS score, 0–10)	7.3 ± 1.5
Duration of symptoms (months)	18.4 ± 6.7
Functional limitations	141 (77.5)
Bilateral CTS	163 (89.6)
Severe hand	
Right	128 (70.3)
Left	42 (23.1)
Equal	12 (6.6)
Nighttime symptoms	156 (85.7)
Occupational exposure	82 (45.1)
History of wrist trauma	27 (14.8)
Family history of CTS	19 (10.4)

Comorbidities were common among the 182 patients, with obesity (76, 41.8%), diabetes mellitus (41, 22.5%), and hypertension (30, 16.5%) being the most prevalent. Other conditions included hypothyroidism (29, 15.9%), rheumatoid arthritis (13, 7.1%), cervical spine disorders (8, 4.4%), and osteoarthritis (5, 2.7%). Less frequent comorbidities, such as coronary artery disease, dyslipidemia, and asthma, were present in fewer than four (2%) patients. Notably, the high prevalence of obesity and diabetes mellitus may be linked to their respective pathophysiological mechanisms. Obesity likely contributes to CTS through increased adipose deposition in the carpal tunnel, elevating intracarpal pressure. Diabetes mellitus, through microvascular damage, nerve ischemia, and glycation-related connective tissue changes, may exacerbate CTS risk and severity. These findings highlight the significant burden of metabolic and inflammatory conditions in CTS patients (Table 2).

**Table 2:** Distribution of comorbidities among CTS patients CTS: carpel tunnel syndrome, BMI: body mass index

Comorbidity	Frequency (%)
Diabetes mellitus	41 (22.5)
Hypertension	30 (16.5)
Obesity (BMI ≥ 30 kg/m²)	76 (41.8)
Hypothyroidism	29 (15.9)
Rheumatoid arthritis	13 (7.1)
Cervical spine disorders	8 (4.4)
Osteoarthritis	5 (2.7)
Coronary artery disease	4 (2.2)
Dyslipidemia	3 (1.6)
Asthma	2 (1.1)
Cholecystitis/cholelithiasis	2 (1.1)
Others	21 (11.5)

Electrodiagnostic studies showed prolonged distal motor latency and greater reduction in sensory nerve conduction velocity in the right hand, with values of 5.3 ± 0.9 ms and 34.9 ± 3.5 m/s, respectively, compared to the left hand, which had 5.1 ± 0.8 ms and 35.6 ± 3.8 m/s, respectively. Similarly, the sensory nerve action potential amplitude was lower in the right hand (9.8 ± 2.7 μV) than in the left (10.2 ± 2.5 μV). Moderate CTS was the most common severity grade, affecting 94 (51.6%) right hands and 73 (40.1%) left hands. Severe CTS was more frequent in the right hand (35, 19.2%) than the left (21, 11.5%), whereas mild CTS was more common in the left hand (72, 39.6%) than the right (50, 27.5%). Normal findings were observed more frequently in the left hand (16, 8.8%) than in the right (3, 1.6%). The greater severity observed in the right hand may be attributed to higher functional demand and repetitive occupational strain, particularly in right-hand-dominant individuals. These findings highlight the role of occupational and functional factors in CTS severity patterns (Table 3).

**Table 3:** Electrodiagnostic findings and severity of CTS in right and left hands CTS: carpel tunnel syndrome, SD: standard deviation

Variables	Right hand	Left hand
	Frequency (%)/mean ± SD	
Electrodiagnostic findings		
Distal motor latency (ms)	5.3 ± 0.9	5.1 ± 0.8
Sensory nerve conduction velocity (m/s)	34.9 ± 3.5	35.6 ± 3.8
Sensory nerve action potential (μV)	9.8 ± 2.7	10.2 ± 2.5
CTS severity		
Mild	50 (27.5)	72 (39.6)
Moderate	94 (51.6)	73 (40.1)
Severe	35 (19.2)	21 (11.5)
Normal	3 (1.6)	16 (8.8)

Among the study subjects, 135 (74.2%) received conservative management, while 47 (25.8%) underwent surgical intervention. The findings highlight a strong association between metabolic and occupational factors and severe CTS. Diabetes mellitus was a major risk factor, with 41 (22.5%) patients having diabetes and an adjusted OR of 7.8 (p < 0.001) for developing severe CTS. Obesity was present in 76 (41.8%) patients and significantly increased the risk of severe CTS (adjusted OR: 3.5, p = 0.002). Repetitive occupational tasks were strongly linked to severity, with 82 (45.1%) individuals engaged in such activities having an adjusted OR of 5.4 (p < 0.001). This underscores the impact of chronic strain on the median nerve. Hypothyroidism, observed in 29 (15.9%) patients, was associated with a nearly fivefold higher risk of severe CTS (adjusted OR: 4.7, p = 0.004), indicating the role of systemic conditions in exacerbating nerve compression. Logistic regression analysis was adjusted for potential confounders such as age, sex, and occupational exposure to improve the reliability of the reported ORs. These findings emphasize the interplay of metabolic comorbidities and occupational strain in CTS severity, highlighting the need for targeted interventions and preventive strategies for at-risk populations. Patients with identified risk factors may benefit from closer monitoring and early management to prevent symptom progression (Table 4).

**Table 4:** Logistic regression analysis of risk factors associated with CTS severity * p-value is statistically significant OR: odds ratio, CI: confidence interval, CTS: carpal tunnel syndrome

Risk factor	Severe (n = 35)	Not severe (n = 147)	OR (95% CI)	Adjusted OR	p-value
Diabetes mellitus (n = 41)	21 (60.0)	20 (13.6)	9.26 (4.2-20.3)	7.8	< 0.001*
Obesity (n = 76)	24 (68.6)	52 (35.4)	3.98 (1.9-8.5)	3.5	0.002*
Repetitive occupational tasks (n = 82)	27 (77.1)	55 (37.4)	5.61 (2.6-12.1)	5.4	< 0.001*
Hypothyroidism (n = 29)	13 (37.1)	16 (10.9)	4.67 (1.9-11.3)	4.7	0.004*

## Discussion

The present retrospective analysis provides a comprehensive overview of the clinical profile, demographics, and risk factors associated with CTS in an Omani cohort over a two-year period. Most CTS patients were female (172, 94.5%), with a mean age of 47.5 ± 10.3 years. This finding aligns with global and regional studies consistently reporting a higher prevalence of CTS among females, attributed to hormonal differences, smaller carpal tunnel dimensions, and occupational factors [13,14]. Housewives constituted the largest occupational group (139, 76.4%), reflecting the sociocultural context of Oman, where women predominantly manage household duties. Repetitive, hand-intensive tasks such as cooking, cleaning, and sewing may contribute to sustained median nerve compression. Additionally, cultural norms may lead to delayed healthcare-seeking behavior, potentially exacerbating symptom severity. A study by Chacko et al. reported similar trends, with 85 (85%) female and 15 (15%) male patients, where 77 (77%) had bilateral involvement, and 23 (23%) had unilateral CTS [15]. A smaller proportion of patients in our cohort were engaged in professions such as nursing (12, 6.6%), teaching (9, 4.9%), and computer-related work (5, 2.7%), indicating that occupational exposure to repetitive hand movements remains a significant risk factor.

The mean symptom duration was 18.4 ± 6.7 months, and the mean pain severity (visual analog score) was 7.3 ± 1.5, highlighting the chronic nature of CTS in this population. Prolonged symptom duration may significantly impair daily activities such as cooking, driving, and writing, which are common in this population. Additionally, the persistent discomfort associated with CTS has been linked to anxiety, depressive symptoms, and sleep disturbances, further impacting mental well-being and reducing overall quality of life [16,17]. These factors may also delay healthcare-seeking behavior, as individuals may initially adopt self-management strategies before seeking medical attention. One hundred forty-one (77.5%) patients reported functional limitations, and bilateral involvement was observed in 163 (89.6%) cases, underscoring the debilitating impact of CTS on daily activities.

Comorbidity analysis revealed that obesity was present in 76 (41.8%) patients and diabetes mellitus in 41 (22.5%). Obesity is a well-documented risk factor, as increased adipose tissue within the carpal tunnel can exert compressive forces on the median nerve, leading to CTS [18]. Additionally, obesity has been linked to altered metabolic profiles, which may promote systemic inflammation and exacerbate neural damage. Similarly, diabetes is associated with neuropathic changes and microvascular complications that contribute to nerve dysfunction, with emerging evidence suggesting that diabetic patients experience faster CTS progression and poorer treatment outcomes [19]. Other notable comorbidities included hypertension in 30 (16.5%), hypothyroidism in 29 (15.9%), and rheumatoid arthritis in 13 (7.1%). Hypothyroidism can cause mucopolysaccharide deposition, leading to perineural edema and median nerve compression, while rheumatoid arthritis is linked to CTS due to chronic synovial inflammation [20]. The presence of multiple metabolic and inflammatory conditions may act synergistically, worsening the prognosis and reducing the efficacy of conservative treatments in some patients.

Cervical spine disorders were observed in eight (4.4%) patients and osteoarthritis in five (2.7%), highlighting the potential overlap of symptoms between CTS and other musculoskeletal conditions, necessitating careful differential diagnosis. These findings align with previous studies that report varying prevalence rates of comorbid conditions based on geographic and demographic factors [21,22].

Electrodiagnostic studies revealed a mean distal motor latency of 5.1 ± 0.8 ms in the left hand and 5.3 ± 0.9 ms in the right hand, with sensory nerve conduction velocities of 35.6 ± 3.8 m/s and 34.9 ± 3.5 m/s, respectively. The greater reduction in sensory nerve conduction velocity and prolonged motor latency in the dominant (right) hand reflects the impact of sustained occupational strain. Dominant-hand involvement is often associated with more pronounced functional impairment, as repetitive hand-intensive activities can accelerate nerve compression and inflammatory responses, contributing to more severe clinical manifestations [23]. Severity grading showed moderate CTS in 94 (51.6%) right hands and 73 (40.1%) left hands. Mild CTS was more common in the left hand (73, 39.6%), while severe CTS predominantly affected the right hand (35, 19.2%). Normal findings were more frequent in the left hand (16, 8.8%) compared to the right (3, 1.6%), suggesting a higher burden of severe disease in the dominant hand. These observations align with studies reporting similar severity patterns, emphasizing the role of hand dominance and occupational strain in CTS pathophysiology [24,25].

In our study, 135 (74.2%) participants opted for conservative management, while 47 (25.8%) underwent surgical intervention. These findings reflect a preference for non-surgical approaches, despite global evidence indicating the superiority of surgical decompression for sustained symptom relief and functional recovery [26,27]. The preference for conservative treatment may stem from cultural beliefs, fear of surgical intervention, or limited awareness regarding the long-term benefits of surgical management.

Multivariate analysis identified diabetes mellitus (41, 22.5%) (adjusted OR: 7.8, p < 0.001), obesity (76, 41.8%) (adjusted OR: 3.5, p = 0.002), repetitive occupational tasks (82, 45.1%) (adjusted OR: 5.4, p < 0.001), and hypothyroidism (29, 15.9%) (adjusted OR: 4.7, p = 0.004) as significant predictors of severe CTS. These findings align with studies highlighting similar risk factors across diverse populations [28-30]. The strong association between repetitive occupational tasks and severe CTS underscores the need for ergonomic interventions and workplace modifications to reduce the disease burden. Educational initiatives promoting early symptom recognition and encouraging timely healthcare-seeking behavior could further improve outcomes.

Limitations

This study has certain limitations. It relied on previously recorded data as a retrospective analysis, which may have inherent biases or missing information. The single-center design may limit the generalizability of the findings to other populations or regions, particularly given the sociocultural context unique to Oman. Additionally, potential selection bias may have influenced participant characteristics, as patients attending this tertiary care center may represent individuals with more severe or prolonged symptoms. Moreover, while multivariate analysis was conducted to adjust for confounding factors, residual confounders related to lifestyle, dietary patterns, or unmeasured occupational factors may have influenced the observed associations. Lastly, the absence of long-term follow-up data restricted the evaluation of sustained outcomes after treatment. Future multicenter prospective studies with larger sample sizes are recommended to validate and expand upon these findings.

## Conclusions

This study highlights the significant impact of CTS on the Omani population, with distinct demographic, clinical, and electrodiagnostic profiles. The high prevalence of comorbid conditions such as obesity and diabetes underscores the need for multidisciplinary approaches to management. While surgical intervention remains the treatment of choice for severe CTS, conservative treatments play a crucial role in managing milder cases or serving as interim management before surgery. Future research should focus on prospective, multicenter studies involving diverse populations, with long-term follow-up data, to better evaluate treatment outcomes and inform preventive strategies targeting modifiable risk factors such as obesity and occupational strain.
